# Orthokeratology to Control Myopia Progression: A Meta-Analysis

**DOI:** 10.1371/journal.pone.0124535

**Published:** 2015-04-09

**Authors:** Yuan Sun, Fan Xu, Ting Zhang, Manli Liu, Danyang Wang, Yile Chen, Quan Liu

**Affiliations:** 1 Zhongshan Ophthalmic Center, Sun Yat-sen University, Guangzhou, Guangdong Province, China; 2 Department of Ophthalmology, People's Hospital of Guangxi Zhuang Autonomous Region, Nanning, Guangxi Province, China; Rush University Medical Center, UNITED STATES

## Abstract

**Objective:**

To evaluate the clinical treatment effects of orthokeratology to slow the progression of myopia.

**Methods:**

Several well-designed controlled studies have investigated the effects of orthokeratology in school-aged children. We conducted this meta-analysis to better evaluate the existing evidence. Relevant studies were identified in the Medline and Embase database without language limitations. The main outcomes included axial length and vitreous chamber depth reported as the mean ± standard deviation. The results were pooled and assessed with a fixed-effects model analysis. Subgroup analyses were performed according to geographical location and study design.

**Results:**

Of the seven eligible studies, all reported axial length changes after 2 years, while two studies reported vitreous chamber depth changes. The pooled estimates indicated that change in axial length in the ortho-k group was 0.27 mm (95% confidence interval [CI]: 0.22, 0.32) less than the control group. Myopic progression was reduced by approximately 45%. The combined results revealed that the difference in vitreous chamber depth between the two groups was 0.22 mm (95% confidence interval [CI]: 0.14, 0.31). None of the studies reported severe adverse events.

**Conclusion:**

The overall findings suggest that ortho-k can slow myopia progression in school-aged children.

## Introduction

Myopia is the most common ocular disorder in humans. In the past 50 years, its prevalence has rapidly risen to 80–90% in some East Asian countries [1]. This increased prevalence is not restricted to Asia; the rate of myopia is also increasing in North America, albeit more slowly [2]. A high degree of myopia has been related with the onset of some blinding pathologies, including macular degeneration, retinal detachment, choroidal neovascularization, cataracts and glaucoma [3]. The extensive and growing prevalence, relevant ocular morbidity, and substantial costs associated with myopia have made it a significant public health issue.

Walline et al. compared various interventions including multifocal lenses, rigid and soft contact lenses, timolol drops, and muscarinic receptor antagonists and found that the latter was the most effective in slowing myopia progression [4]. However, they failed to assess the effectiveness of orthokeratology (ortho-k); this technique involves reshaping the epithelium to correct ametropia and was first described in the early 1960s [5]. Its efficacy was improved by the introduction of new materials and a reverse geometry lens design. In 2002, the U.S. Food & Drug Administration approved an overnight-wear contact lens by Paragon Vision Sciences, which led to improved patient compliance [6].

Due to the studies with large sample sizes were not available, we reviewed a number of relevant trials to obtain more precise estimates of the myopic control following overnight use of ortho-k lenses in pediatric subjects.

## Materials and Methods

### Search Strategy

Two researchers (YS and FX) queried the Medline and Embase databases to identify relevant studies using the following keywords: “corneal reshaping”, “CRT”, “OK”, “orthokeratology”, “ortho-k”, “ametropia” and “myopia”. The final search was performed on January 25th, 2014. The results were not limited by language. We hand-searched the references of the relevant articles and reviews to identify additional studies that may have been missed.

### Study Inclusion Criteria

Considering the paucity of available randomized clinical studies, well-designed controlled studies were also included in the current meta-analysis if they met the following inclusion criteria: (1) measurement of axial length (AL) between the baseline and end of the study, (2) at least two comparison groups (intervention and control), and (3) the follow-up period was more than 1 year.

### Study Selection and Data Extraction

Two researchers (YS and FX) separately scanned the identified titles and abstracts to determine if they might meet the inclusion criteria. The full texts were subsequently retrieved if the articles met the criteria or if that could not be determined based on the titles and abstracts. Inconsistencies were resolved through discussion with a third researcher (TZ). When multiple trials from the same study population were available, the publication with more complete data that was a better fit with our research objective was included. The following information was collected for each study: first author’s name, country or area of the research, year of publication, and AL and vitreous chamber depth (VCD) at the baselines and endpoints.

### Quality Assessment

Two authors (YS and FX) separately performed the quality assessments of all the included studies. We used the Jadad scale to access the evidence quality of the randomized controlled trials (RCTs), with scores of 0 and 5 indicating the lowest and highest qualities, respectively [7]. The checklist included methods of randomization, masking, and withdrawal. For the remaining observational controlled trials (CTs), a methodological index for non-randomized studies (MINORS) was used to perform the assessments. According to MINORS, the scores were 0 and 24 for the lowest and highest levels of evidence, respectively [8]. Additionally, we used the Grading of Recommendations, Assessment and Evaluation (GRADE) system to evaluate outcome quality [9–11]. Five items including limitations, inconsistency, indirectness, imprecision, and publication bias were assessed to decrease the weight of evidence, which was estimated by four grades: high, moderate, low or very low. As our eligible studies contained both RCTs and CTs, we selected criteria that could be applied to both. Because our datasets included those from RCTs, we did not consider automatic downgrading, which is only performed for datasets including observational trials; therefore we also did not consider the criteria for upgrading. The evidence profile table was created using the GRADEpro 3.6 software.

### Statistic Analysis

All extracted data were imported into a database, and all statistical analyses were performed using STATA 11.0 (Stata Corporation, College Station, TX). The weighted mean differences (WMDs) and 95% confidence intervals (CIs) were calculated for each study to assess AL and VCD changes between the two groups (intervention and control). The absolute changes (Means and standard deviations) in AL and VCD were used to calculate the WMDs. Statistical heterogeneity was evaluated using the chi-square-based Q statistic and the I^2^ statistic. The Q statistic was considered significant if P < 0.1, and the WMD was pooled according to the fixed-effects model. If there was significant heterogeneity, a random effects model was used and one-way sensitivity analyses were performed, removing each study in turn to evaluate the influence of each individual trial on the pooled outcomes. Subgroup analyses were carried out according to the different geographical locations and study designs.

## Results

### Search Results and Study Characteristics

**Fig 1:**
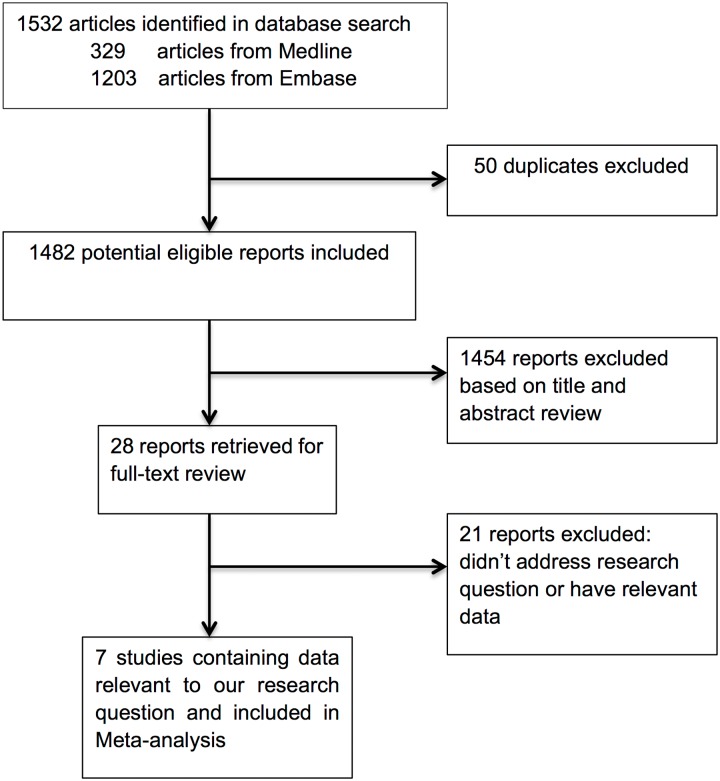
The flowchart of study selection.

The initial search identified a total of 1532 articles: 329 and 1203 in Medline and Embase, respectively. We excluded 50 duplicate papers and 1454 articles based on the titles and abstracts; 28 reports were identified and retrieved for full-text review. Ultimately, seven eligible studies were included in the current meta-analysis (Fig 1) [12–18].

The characteristics of the seven included articles are listed in Table 1. These studies included a total of 546 individuals (age range from 6 to 16 years old), and 435 subjects completed the 24-month follow-up visit. The drop-out rate ranged from 12.4% to 46.2%. Only two trials were randomized, and the studies employed different recruitment criteria. For example, Charm et al. focused on the myopic control effect of ortho-k for highly myopic children (myopia at least 5.00D) and Chen et al. concentrated on myopic children with moderate astigmatism (1.25D to 3.5D) [15, 17]. The weight of the evidence was considered moderate according to the GRADE system (Table 2).

**Table 1:** Main characteristics of studies included in this meta-analysis.

Study	Country or Area	Age (year)	Design	Control	Inclusion criteria	Instrument	Dropout	Quality
Cho 2005	Hong Kong	7–12	CT	Spectacle	-0.25DS to -4.50DS, less than 2.00DC	A-scan	19%	16 (minors)
Walline 2009	USA	8–11	CT	Soft contact lens	-0.75DS to -4.00DS, less than 1.00DC	A-scan	30%	16 (minors)
Kakita 2011	Japan	8–16	CT	Spectacle	SE between -0.5D and -10.0D	IOLmaster	12%	19 (minors)
Cho 2012	Hong Kong	6–10	RCT	Spectacle	-0.50DS to -4.00DS, less than 1.25DC	IOLmaster	24%	3 (Jadad)
Santodomingo 2012	Spain	6–12	CT	Spectacle	-0.75DS to -4.00DS, less than 1.00DC	IOLmaster	13%	18 (minors)
Charm 2013	Hong Kong	8–11	RCT	Spectacle	SE at least -5.75D	IOLmaster	46%	3 (Jadad)
Chen 2013	Hong Kong	6–12	CT	Spectacle	-0.5DS to -5.0DS, 1.25DC to 3.50DC	IOLmaster	28%	22 (minors)

**Table 2:** Grading of Recommendations, Assessment, and Evaluation (GRADE) ranking for evidence of two outcomes.

No of studies	Risk of bias	Inconsistency	Indirectness	Imprecision	Other considerations	Ortho-k	Control	Mean Difference	Quality	Importance
7 studies^1^	Serious^3^	No serious inconsistency	No serious indirectness	No serious imprecision	None	218	217	0.27 (CI: 0.32 to 0.22)	Moderate	Critical
2 studies^2^	Serious^3^	No serious inconsistency	No serious indirectness	No serious imprecision	None	63	63	0.22 (CI: 0.31 to 0.14)	Moderate	Critical

^1^ Axial length (follow-up mean 2 years; better indicated by lower values).

^2^ Vitreous chamber length (follow-up mean 2 years; better indicated by lower values).

^3^ Most of studies did not use blinding method.

### Change in AL

**Fig 2:**
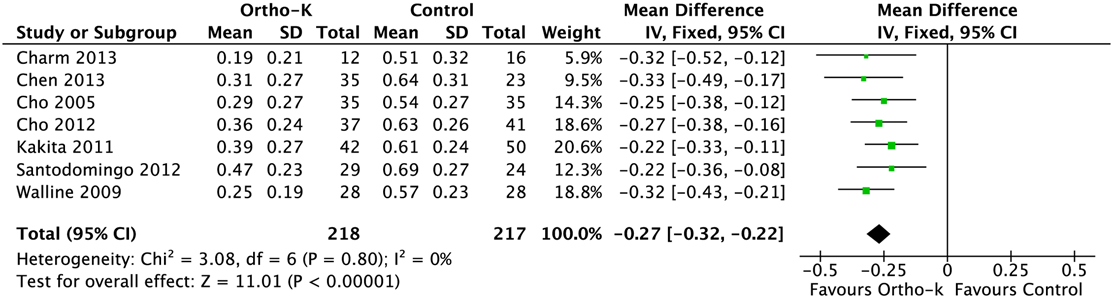
Forest plot of the treatment effect of orthokeratology on change in axial length. CI = confidence interval.

Overall, the combined results showed that the mean AL of the 218 subjects in the ortho-k group was 0.27 mm (95% CI: 0.22, 0.32) less than that of 217 subjects in the control group after two years (Fig 2). There was no statistical heterogeneity between the two groups (P = 0.80, I^2^ = 0%). We also performed subgroup analyses according to the study design and geographical location. Of the seven included studies, two randomized controlled trials were analyzed using a fixed-effects model. The WMD in the change of AL was 0.28 mm (95% CI 0.19, 0.38; P = 0.66, I^2^ = 0%). The remaining five observational studies were homogeneous (P = 0.59, I^2^ = 0%); the WMD in AL change was 0.26mm (95% CI 0.21, 0.32). The results of the five studies conducted in Asia suggested a statistically significant difference between the ortho-k and control groups (0.26 mm, 95% CI: 0.21, 0.32, p = 0.78%, I^2^ = 0%), which was similar with the trials conducted outside Asia (0.28 mm, 95% CI: 0.19, 0.37, P = 0.26, I^2^ = 20%).

### Change in VCD

**Fig 3:**
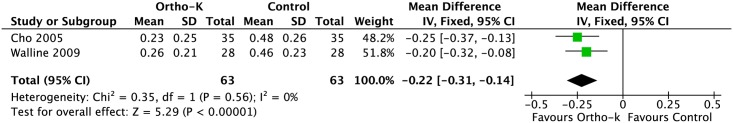
Forest plot of the treatment effect of orthokeratology on change in vitreous chamber depth. CI = confidence interval.

The combined results showed that there was a significant difference in VCD between the ortho-k and control groups (Fig 3). The WMD with a 95% CI was 0.22 mm (95% CI: 0.14, 0.31). There was no significant between-study heterogeneity (P = 0.56, I^2^ = 0%).

### Adverse Event

No studies reported severe adverse events. The most common ocular health issues were corneal stains and pigmented arcs. All reported corneal stains were mild (no more than grade two) and usually affected the inferior cornea. Cho et al. reported one patient who developed chalazion after 21 months of lens wear [15]. Another study described one subject with corneal opacities; however, his ophthalmologist confirmed that the opacities were probably caused by allergies and were not related to ortho-k [17]. Further more, no keratitis was reported in any of the ortho-k groups.

## Discussion

This results of this meta-analysis illustrate that ortho-k can slow the progression of myopia in school-aged children. The rate of AL elongation was slowed by 0.14 mm per year in the ortho-k group compared to the control group. This corresponds to nearly 45% decrease in myopic progression. The heterogeneity among these seven studies was very small, which indicated that the results were consistent and the CI was precise (95% CI: 0.22, 0.32). The results remained stable, when we separately analyzed the study design and geographical location. Ortho-k decreased myopic progression by 44% and 45% in Asian and non-Asian children, respectively, suggesting that both groups experience similar benefits from ortho-k. However, only two studies were conducted outside Asia, and more investigations are needed to confirm these results.

Ortho-k can shift myopia to emmetropia by flattening the cornea within a short period of time after beginning treatment [19]. The measurement of the refractive error does not actually reflect the genuine treatment effects of ortho-k. The method to measure refractive changes in the identified studies was not comparable, and the viewpoint on how long treatment was needed was not uniform. Meanwhile, eyeball expansion has been associated with the onset of blinding pathologies. Therefore we chose the change in AL as the main outcome.

Cheung at el. Claimed that the anterior segment length did not change during the lens-wearing period [20]. According to our findings, VCD elongation was slowed by nearly 0.11 mm per year in the ortho-k group compared to the control group, which corresponded to the change in AL elongation. This supports the hypothesis that the ability of ortho-k to lessen AL elongation is largely due to its effect on slowing the growth of the vitreous chamber.

Kakita et al. found that the rate of AL elongation in the ortho-k group was only correlated with the spherical equivalent refractive error at baseline in highly myopic children [14]. Additionally, Cho at el. demonstrated that there was a relationship between AL and the age, but no relationship was observed between AL and gender, initial myopia, initial astigmatism, initial corneal shape, or initial corneal toricity [17,18]. Chen et al. separately analyzed the effects of ortho-k on large and small pupils and found that AL elongation in the ortho-k group was slower for subjects with larger pupil size (above average) [21].

Atropine is a major alternative treatment to ortho-k for progressive myopia. Previous studies have examined the effects of various concentrations of atropine from 0.01% to 1%. The effects varied from 59% to 77% and were dose dependent [4,22,23]. It seemed that the percentage reduction was higher than ortho-k as determined in the current met-analysis. However, the adverse events and myopia recurrence after cessation have limited the clinical application of atropine. The ATOM2 study indicated that a lower atropine concentration was associated with less myopia rebound after cessation than a high concentration [23]. Clinical investigations assessing the efficacy of low concentrations of atropine are currently being conducted. Few studies have measured the myopic progression after cessation of ortho-k treatment, in part because most of the children wearing ortho-k were not willing to stop the treatment and go back to wearing glasses. However, studies extending at least 1 year beyond treatment cessation are needed to investigate whether the refraction would rebound after stopping ortho-k, as has been reported for atropine. Measurements of refractive change after long-term cessation would also be informative. Studies with longer follow-up periods should be conducted to explore the long-term effects of ortho-k.

During the years 2001 to 2008, more than 100 cases of infectious keratitis associated with ortho-k had been reported, including bacteria and acanthamoeba [24]. For the including studies, no keratitis was noted in the ortho-k groups. Only one child developed corneal opacities in both eyes. However, these were not considered to be directly caused by ortho-k; the authors attributed them to allergies and a delayed diagnosis [17]. No serious adverse effects were reported in any of the patients in the included studies. However, the sample size of the current meta-analysis was not large enough to thoroughly evaluate the safety of ortho-k. Because the target population were children, a high level of vigilance is necessary during the entire wearing period; thorough care and informed parents are required to ensure safety and good outcomes.

### Limitations of the Meta-analysis

There are several limitations in this meta-analysis. Firstly, two studies used historical comparisons; the control and intervention groups were not treated and assessed during the same time period [12,13]. Secondly, no studies reported the use of double-blinding design, and it was hard to hide the allocation for researchers who performed the examinations. That could lead to an overestimate the myopic control effect of ortho-k. Thirdly, we did not assess for a possible publication bias. Although this kind of bias could be tested using funnel plots and statistical methods (Begg’s and Egger’s test), these results might not be conclusive with such a small number of trials. However, we employed a broad search strategy and meticulous identification for the databases and reference lists to minimize the likelihood of any publication bias. Moreover, there were only two studies measuring VCD changes included in this meta-analysis. Further researches are necessary to obtain a more accurate estimate.

In summary, ortho-k slows myopia progression in school-aged children. However, no studies had assessed a large number of participants. Further research should be conducted to assess the effects of ortho-k on myopic control compared with other interventions, such as atropine; RCTs would be especially informative in clarifying the effects of this treatment.

## Supporting Information

S1 PRISMA 2009 Checklist (DOC)
