# Correction: Orthokeratology to Control Myopia Progression: A Meta-Analysis

**DOI:** 10.1371/journal.pone.0130646

**Published:** 2015-06-11

**Authors:** Yuan Sun, Fan Xu, Ting Zhang, Manli Liu, Danyang Wang, Yile Chen, Quan Liu

There is an error in affiliation 1 for authors Yuan Sun, Fan Xu, Ting Zhang, Manli Liu, Danyang Wang, Yile Chen, and Quan Liu. Affiliation 1 should be: State Key Laboratory of Ophthalmology, Zhongshan Ophthalmic Center, Sun Yat-sen University, Guangzhou, Guangdong Province, China.
